# Primary melanoma in the bronchus

**DOI:** 10.1002/ccr3.5192

**Published:** 2021-12-09

**Authors:** Akina Nigi, Hirokazu Toyoshima, Shigeto Kondo, Motoaki Tanigawa

**Affiliations:** ^1^ Department of Respiratory Medicine Japanese Red Cross Ise Hospital Ise Japan; ^2^ Department of Infectious Diseases Japanese Red Cross Ise Hospital Ise Japan

**Keywords:** bronchoscopy, primary malignant melanoma of the lung, typical pigmentation

## Abstract

Primary malignant melanoma of the lung (PMML) is an extremely rare tumor with a dismal prognosis. Distinguishing PMML from metastatic melanoma of the lung can be difficult without an established treatment protocol for advanced PMML. We present a case of immunotherapy‐treated PMML wherein the patient died 3 months after treatment.

## Graphical abstract

Bronchoscopy facilitates diagnosis of primary malignant melanoma of the lung (PMML), with typical pigmentation, as it mainly occurs in the central lung areas. Clinicians should accurately distinguish PMML from skin melanoma for proper management.

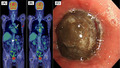

## 1 CLINICAL IMAGE

**FIGURE 1:**
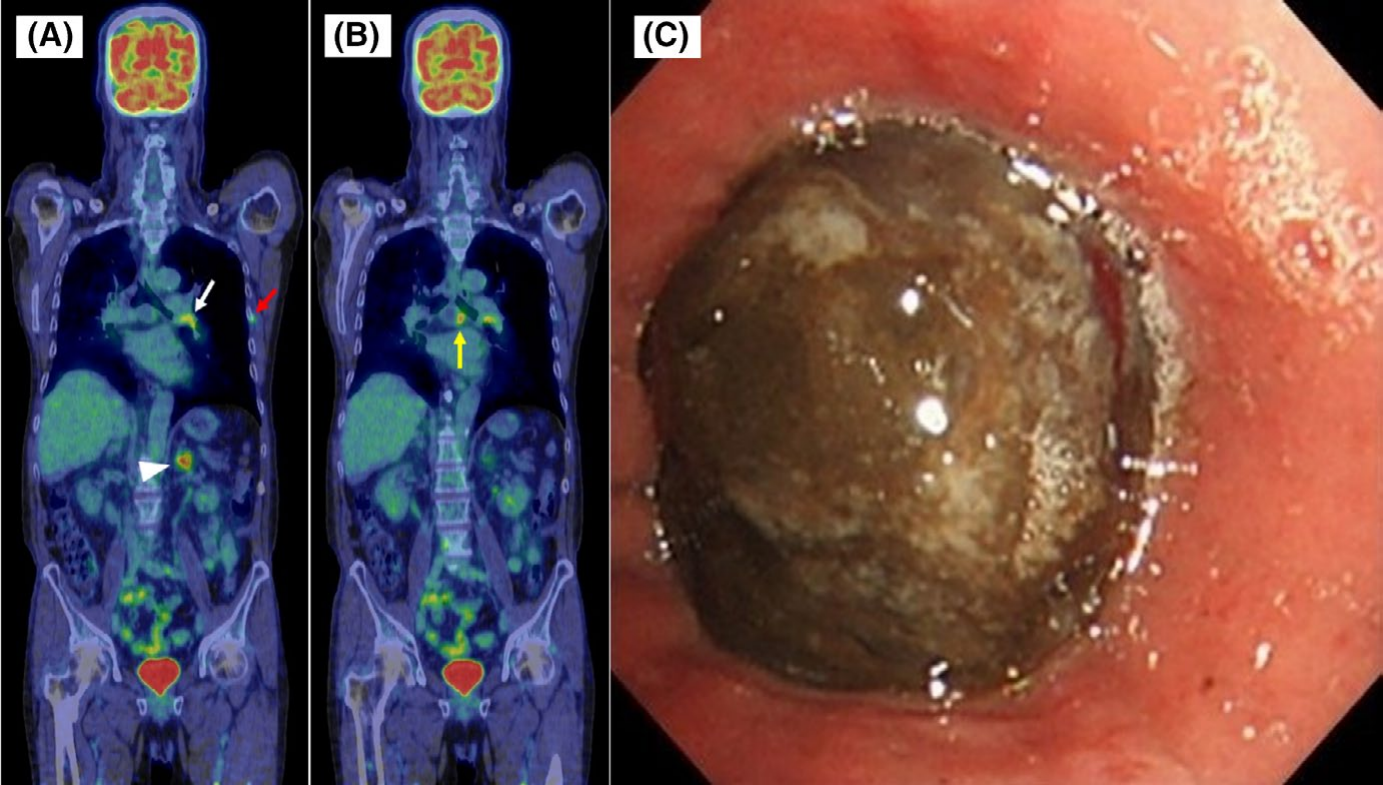
18F‐fluorodeoxyglucose positron emission tomography‐computed tomography imaging shows a polypoid lesion (white arrow) in the left bronchus (A) with metastasis to the left adrenal gland (white arrowhead), rib (red arrow) (A), and mediastinal lymph nodes (yellow arrow) (B). Bronchoscopy shows a pigmented mass in the left main bronchial lumen (C)

**FIGURE 2:**
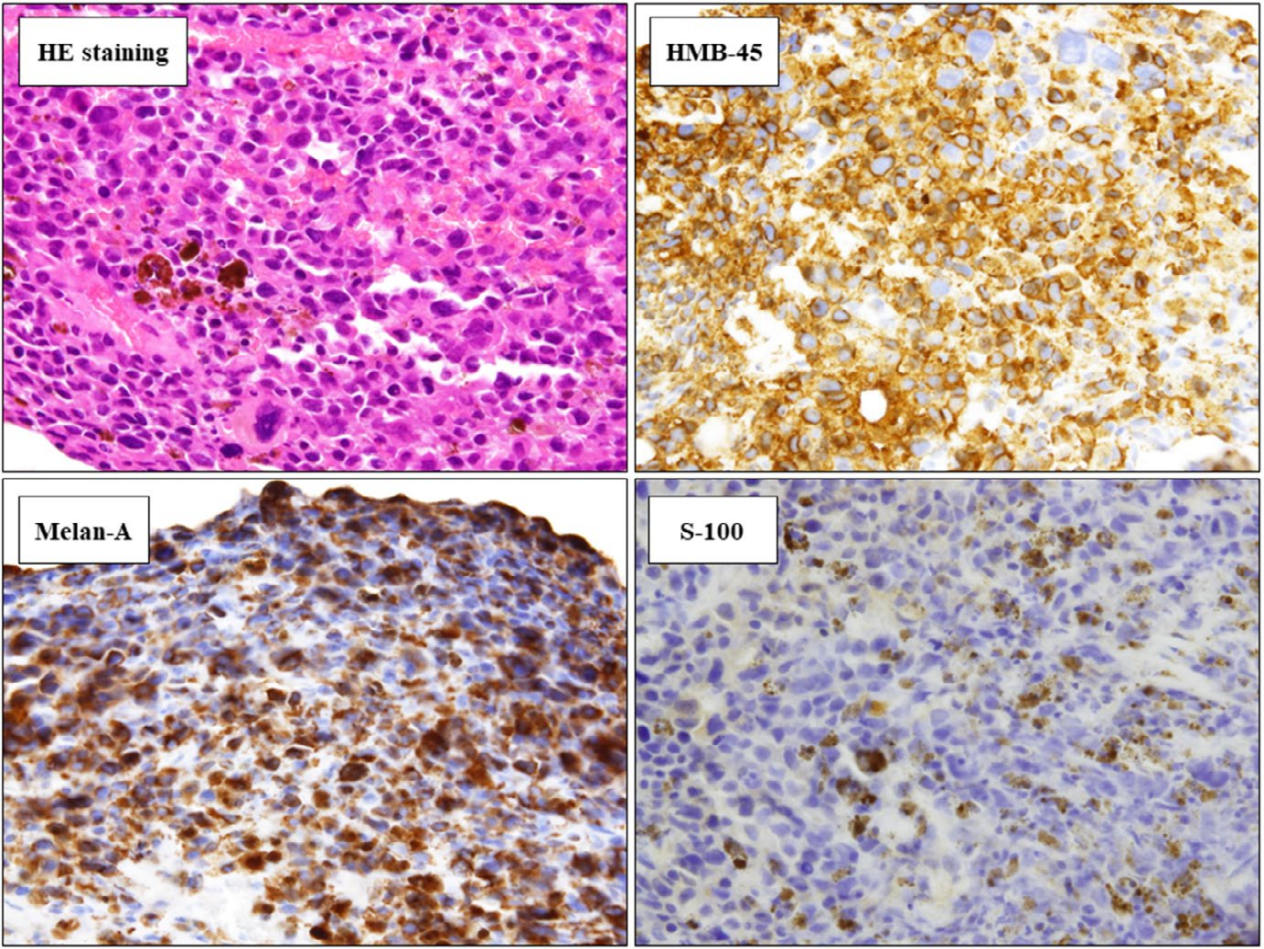
Hematoxylin and eosin staining of the biopsy specimen shows pleomorphic cells with brown pigmentation and lack of cellular maturation. On immunohistochemical staining, the specimen tested positive for the Human Melanoma Black (HMB‐45) antibody, Melan‐A, and S‐100 protein antibody

A 71‐year‐old man presented with a 1‐month history of chest pain and progressive dyspnea. Physical examination revealed left lung wheezes without desaturation at rest. 18F‐fluorodeoxyglucose positron emission tomography‐computed tomography showed a polypoid lesion in the left bronchus and multiple mediastinal lymph nodes (Figure 1A,B). Bronchoscopy revealed a left main bronchus intraluminal pigmented mass (Figure 1C). Transbronchial biopsy specimen stained with hematoxylin‐eosin revealed poorly circumscribed immature brown‐pigmented cells; immunohistochemistry revealed positive staining for Human Melanoma Black‐45, Melan‐A, and S‐100 (Figure 2). V‐raf murine sarcoma viral oncogene homolog B1 mutation was not detected. Low programmed cell death‐ligand 1 expression was noted. Whole‐body examination revealed no other malignancy. The patient was intraoperatively diagnosed with advanced primary malignant melanoma of the lung (PMML), stage Ⅳb (T3N2M1c), and treated with pembrolizumab. He died after 3 months.

Bronchoscopy is necessary to diagnose PMML with characteristic pigmentation because it mainly occurs in central lung areas.^1^ Both immunotherapy and molecular targeted therapy are less effective for PMML than for skin melanoma^1^; therefore, given the rarity of PMML, in the absence of an established treatment protocol, advanced PMML can be treated using the CheckMate‐066 study regimen.^2^ Clinicians must consider the biological characteristics of PMML for appropriate clinical management.

## CONFLICTS OF INTERESTS

None.

## AUTHOR CONTRIBUTION

AN contributed to the clinical management of the patient and drafting of the manuscript. HT contributed to the clinical management of the patient and was involved in study conception; data acquisition and analysis; and supervision, drafting, and critical revision of the manuscript. SK contributed to the clinical management of the patient. MT contributed to the clinical management of the patient and was involved in supervision, drafting, and critical revision of the manuscript. All authors reviewed the final draft of the manuscript and approved its submission.

## ETHICAL APPROVAL

This study was approved by the Institutional Review Board and Ethics Committee of the Japanese Red Cross Ise Hospital (Permission number: ER2020‐104).

## CONSENT

Written informed consent was obtained from the patient for the publication of this case report and the accompanying images. A copy of the written consent is available for review from the Editor‐in‐Chief of this journal on request.

## Data Availability

The data that support the findings of this study are available from the corresponding author upon reasonable request.
